# The Antiproliferative and Apoptotic Effects of a Novel Quinazoline Carrying Substituted-Sulfonamides: In Vitro and Molecular Docking Study

**DOI:** 10.3390/molecules27030981

**Published:** 2022-02-01

**Authors:** Ali S. Alqahtani, Mostafa M. Ghorab, Fahd A. Nasr, Mohammad Z. Ahmed, Abdullah A. Al-Mishari, Sabry M. Attia

**Affiliations:** 1 Department of Pharmacognosy, College of Pharmacy, King Saud University, Riyadh 11451, Saudi Arabia; mahmed4@ksu.edu.sa; 2 Medicinal, Aromatic and Poisonous Plants Research Center, College of Pharmacy, King Saud University, Riyadh 11451, Saudi Arabia; fnasr@ksu.edu.sa (F.A.N.); aalmshari@ksu.edu.sa (A.A.A.-M.); 3 Department of Drug Radiation Research, National Center for Radiation Research and Technology (NCRRT), Egyptian Atomic Energy Authority (EAEA), Nasr City, Cairo 11371, Egypt; 4 Department of Pharmacology and Toxicology, College of Pharmacy, King Saud University, Riyadh 11451, Saudi Arabia; attiasm@ksu.edu.sa

**Keywords:** cell cycle, quinazoline, sulfonamide, molecular docking

## Abstract

In order to investigate for a new effective and safe anticancer drug, we synthesized a novel series of quinazoline containing biologically active substituted-sulfonamide moiety at 3- position **4a**–**n**. The structure of the newly prepared compounds was proved by microanalysis, IR, ^1^H-NMR, ^13^C-NMR and mass spectral data. All the synthesized compounds were evaluated for their in vitro cytotoxic activity in numerous cancer cell lines including A549, HepG-2, LoVo and MCF-7 and normal HUVEC cell line. The two most active compounds **4d** and **4f** were then tested for their apoptosis induction using DNA content and Annexin V-FITC/PI staining. Moreover, apoptosis initiation was also confirmed using RT-PCR and Western blot. To further understand the binding preferences of quinazoline sulfonamides, docking simulations were used. Among the fourteen new synthesized compounds, we found that compounds **4d** and **4f** exerted the strongest cytotoxicity against MCF-7 cells with an IC_50_ value of 2.5 and 5 μM, respectively. Flow cytometry data revealed the ability of compounds **4d** and **4f** to mediate apoptosis and arrest cell cycle growth at G1 phase. Furthermore, RT-PCR and Western blot results suggested that both **4d** and **4f** activates apoptotic cell death pathway in MCF-7 cells. Molecular docking assessments indicated that compounds **4d** and **4f** fit perfectly into Bcl2’s active site. Based on the biological properties, we conclude that both compounds **4d** and **4f** could be used as a new type of anticancer agent, which provides a scientific basis for further research into the treatment of cancer.

## 1. Introduction

**Figure 1:**
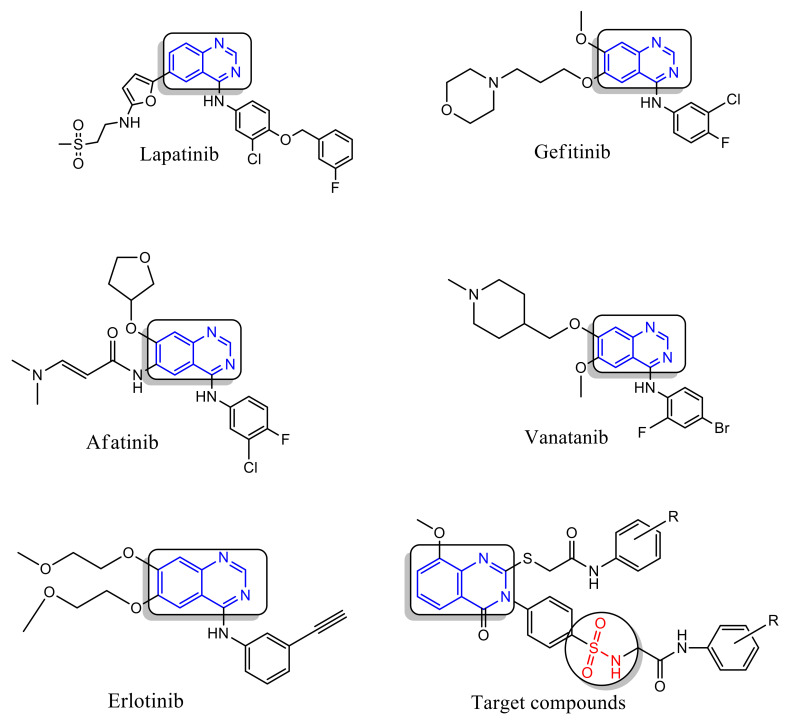
Quinazoline-containing drugs.

Quinazoline and sulfonamide moieties have been identified as classes of chemotherapeutic agents with substantial therapeutic value against solid tumors [1,2,3,4]. Gefitinib (small-molecule inhibitor) being the original quinazoline molecule to be approved as an antitumor agent for treatment of Non-Small Cell Lung Cancer [5,6,7]. Due to the great performances of quinazoline compounds in antitumor applications, the enhancement of new quinazoline candidates as anticancer agents is a promising topic. In fact, several selective derivatives of quinazoline-containing drugs such as lapatinib, erlotinib, and vandetanib have been approved as anticancer drugs [8,9,10] (Figure 1). On the other hand, various biological activities have been attributed to sulfonamide-containing compounds, including antitumor, carbonic anhydrase inhibition, antimalarial, and antimicrobial activities [4,11]. In the new drug design, the development of hybrid derivatives through the combination of several pharmacophores may assign compounds with remarkable biological profiles [12]. Previously, various quinazoline sulfonamide derivatives were evaluated as anticancer agents against numerous cell lines, and many compounds had displayed a promising activity [3,13,14]. Apoptosis evasion is a remarkable feature of most cancer cells; therefore, designing drugs that induce apoptosis has emerged as a promising candidate for anti-cancer optimization [15]. In fact, several previous studies have demonstrated that quinazoline scaffolds had a remarkable antiproliferative capacity against a variety of cancer cells, highlighting their efficiency in the development of apoptosis inducers drugs. Moreover, numerous novel quinazoline-derived compounds which exert effective anticancer activity via apoptosis induction have been synthesized [10,16]. Lapatinib and erlotinib are among these quinazoline-containing drugs that have been demonstrated to exert cell cycle arrest and apoptosis initiation in various cancer cells [17,18]. Consequently, efforts have been in use to synthesize a hybrid of quinazoline–sulfonamide in expectation of a promising synergistic consequence on the inhibition of cancer cell propagation. Although, there are no literature reports on substituted quinazoline–sulfonamide hybrid synthesis and its anticancer activity on human breast cancer cell line and adenocarcinoma cell line. Hybridization has confirmed to be valuable for the synthesis of new antitumor agents and overcoming the problems of the traditionally used drugs [19]. Therefore, sulfonamides were included with quinazolin-4(3H)-ones to obtain more effective candidates. As a continuation of our previous efforts [13,20,21].

In this respect, we designed novel quinazoline derivatives bearing substituted-sulfonamide moiety, these derivatives were subjected to in vitro cytotoxic evaluation against numerous cell lines including A549 (lung), LoVo (colon), HepG2 (liver) and MCF-7 (breast) cancer cells as well as HUVEC (normal) cell line. Moreover, the most active compounds were selected to explore its apoptotic effects against the most responsive cancer cells. Molecular docking was also performed to determine their binding mode and their ability to satisfy the pharmacophoric features required to induce the desired inhibition.

## 2. Results and Discussion

### 2.1. Chemistry

**Scheme 1:**
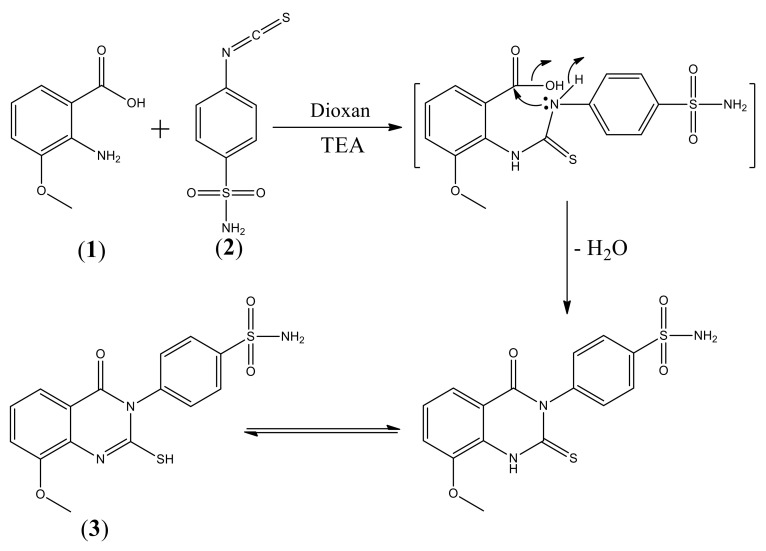
Synthesis of 4-(2-mercapto-8-methoxy-4-oxoquinazolin-3(4*H*)-yl)benzenesulfonamide **3**.

**Scheme 2:**
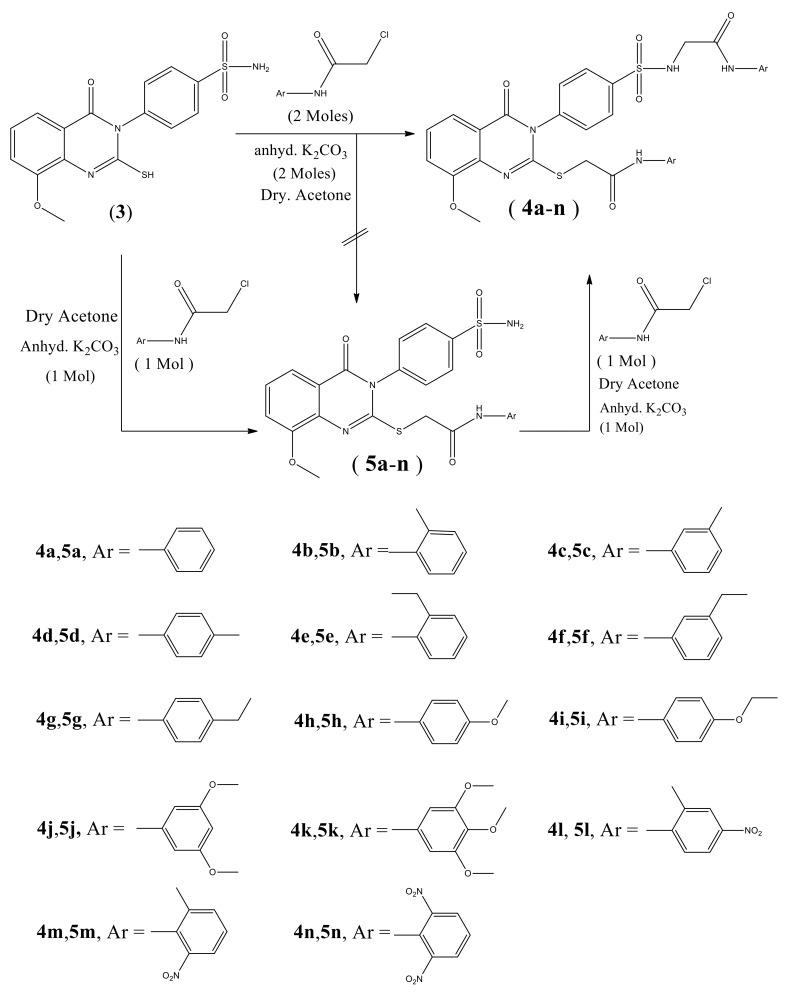
Synthesis of novel quinazolines-substituted sulfonamides **4a**–**n**.

**Scheme 3:**
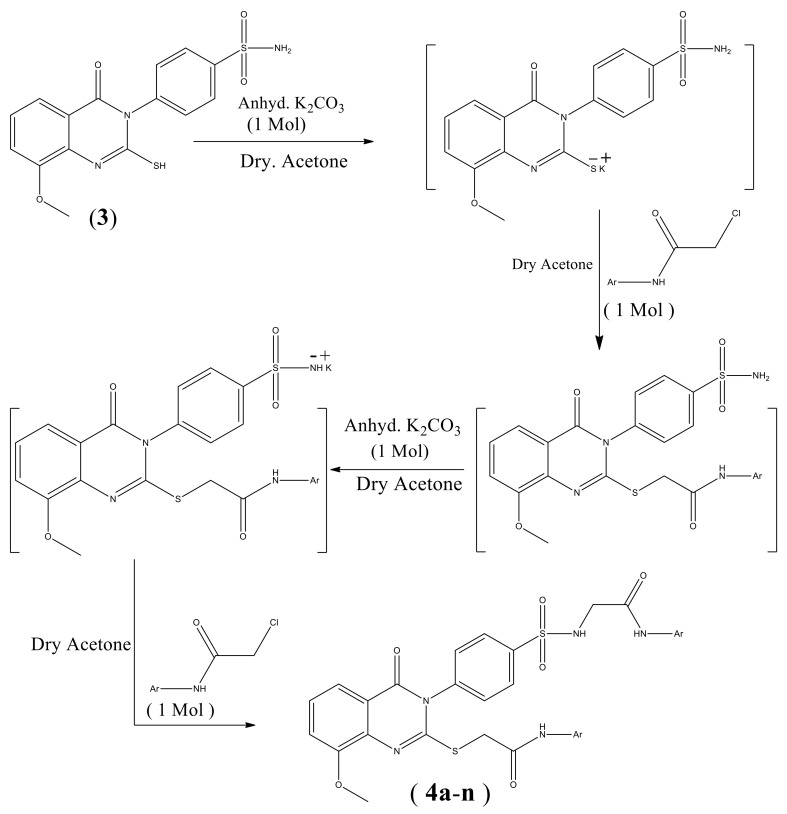
Formation of compounds **4a**–**n**.

Scheme 1 shows the synthesis of quinazoline carrying biologically active substituted- benzenesulfonamide moieties **4a**–**n**. The required strategic starting material 4-(2-mercapto-8-methoxy-4- oxoquinazolin-3(4*H*)-yl) benzenesulfonamide **3** was synthesized in quantitative yield by cyclocondensation of 2-amino-3-methoxybenzoic acid **1** with 4-isothiocyanatobenzenesulfonamide **2** [19], in refluxing 1,4-dioxan containing triethylamine (Scheme 1). IR spectrum of compound **3** exhibited a characteristic band for NH_2_, CH aromatic, C=O, C=N and SO_2_ groups. ^1^H-NMR spectrum revealed signals at 3.9 ppm assigned to OCH_3_ and 12.3 ppm attributed to SH proton. ^13^C-NMR showed signals at 55.7 ppm for OCH_3_ and 160.9 ppm due to C=O of quinazoline ring. The reaction of **3** with 2-chloro-*N*-arylacetamide derivatives was investigated. Thus, reaction of **3** with the excess of 2-chloro-*N*-substituted acetamide (2 mol) in dry acetone containing excess anhydrous K_2_CO_3_ (2 mol) gave the unexpected novel quinazoline incorporated substituted-sulfonamide moieties at 3-position **4a**–**n**, rather than the expected quinazoline- containing free sulfonamide moiety at 3-position **5a**–**n** [22] (Scheme 2). The formation of compounds **4a**–**n** was explained in (Scheme 3). On the other hand, compounds **4a**–**n** were obtained also in good yield via reaction of compounds **5a**–**n** with 2-chloro-*N*-arylacetamide derivatives (1:1 mol) in presence of dry acetone containing (1 mol) anhydrous K_2_CO_3_. All the synthesized compounds **4a**–**n** were established by elemental analysis, IR, ^1^H-NMR, ^13^C-NMR and mass spectral data. IR spectra of **4a**–**n** displayed additional 2NH and 2C=O bands at their assigned regions. ^1^H-NMR spectra of **4a**–**n** revealed signals at 3.7–3.9 ppm assigned to OCH_3_, two singlet signals, one at 3.9–4.1 ppm referring to the SCH_2,_ 4.1–4.3 ppm due to N-CH_2_, and 7.5–8.0 ppm for aromatic protons (AB system), 8.0–8.2 ppm attributed for SO_2_NH proton and 8.8–10.5 ppm for 2NH protons. ^13^C-NMR of **4a**–**n** displayed two signals peculiar to the SCH_2_, NCH_2_ and 3CO carbons. ^1^H-NMR spectrum of **4a** showed singlet signals at 3.8 ppm for OCH_3_, 4.1 ppm for SCH_2_, 4.2 ppm for NCH_2_, 8.0 ppm for SO_2_NH and 10.3 ppm due to 2NH protons. ^13^C-NMR spectrum of compound **4a** revealed signals at 31.6 ppm assigned to SCH_2_, 44.0 ppm attributed to NCH_2_, 56.7 ppm for OCH_3_, 161.4, 165.0, 165.6 ppm for 3C=O. ^1^H-NMR spectrum of **4b** showed signals at 2.1 ppm for 2CH_3_ groups at the *ortho* positions of the phenyl ring*,* 3.8 ppm for OCH_3_, 4.1, 4.3 ppm for 2CH_2_, 8.1 ppm for SO_2_NH and 9.6 ppm due to 2NH protons. ^13^C-NMR spectrum of compound **4b** exhibited signals at 18.1 ppm due to 2CH_3_ protons. ^1^H-NMR spectrum of **4c** revealed signals at 2.3 ppm for 2CH_3_ group at the *meta* positions of the phenyl ring; ^13^C-NMR spectrum of compound **4c** exhibited signals at 21.6 ppm due to 2CH_3_ protons. ^1^H-NMR spectrum of **4d** revealed signals at 2.2 ppm for 2CH_3_ groups at the *para* positions of the phenyl ring. ^13^C-NMR spectrum of compound **4d** showed singlet at 20.9 ppm for 2CH_3_ protons, 28.6, 44.0 ppm due to 2CH_2_ protons. ^1^H-NMR spectrum of **4e** revealed triplets at 1.1 ppm attributed to the 2CH_3_ ethyl, and quartet at 2.5 ppm referring to the 2CH_2_ ethyl at the *ortho* positions of phenyl ring. ^13^C-NMR spectrum of compound **4e** exhibited signals at 14.5 ppm for 2CH_3_ ethyl group, 24.0 ppm due to 2CH_2_ ethyl protons. ^1^H-NMR spectrum of **4f** revealed a triplet at 1.1 ppm attributed to the 2CH_3_ ethyl, and quartet at 2.6 ppm referring to the 2CH_2_ ethyl at the *meta* positions. ^13^C-NMR spectrum of compound **4f** exhibited signals at 15.9 ppm due to 2CH_3_ protons, 28.6 ppm for 2CH_2_ ethyl group. ^1^H-NMR spectrum of **4g** revealed triplets at 1.1 ppm attributed to the 2CH_3_ ethyl, and quartet at 2.6 ppm referring to the 2CH_2_ ethyl at the *para* positions. ^13^C-NMR spectrum of compound **4g** exhibited signals at 16.1 ppm due to 2CH_3_ protons, 28.0 ppm for 2CH_2_ ethyl groups. ^1^H-NMR spectrum of **4h** revealed signals at 3.72, 3.73, 3.80 ppm for 3 OCH_3_ groups. ^13^C-NMR spectrum of compound **4h** exhibited signals at 55.6 ppm due to 3 OCH_3_ protons. ^1^H-NMR spectrum of **4i** revealed triplets at 1.3 ppm attributed to the 2CH_3_ ethoxy groups, and quartet at 4.0 ppm referring to the 2CH_2_ ethoxy groups at the *para* positions. ^13^C-NMR spectrum of compound **4i** exhibited signals at 15.1 ppm for 2CH_3_ ethoxy groups, 63.5 ppm due to 2CH_2_ ethoxy groups. ^1^H-NMR spectrum of **4j** revealed signals at 3.81, 3.84, 3.90 ppm for 5 OCH_3_ groups. ^13^C-NMR spectrum of **4j** exhibited signals at 56.3 ppm due to 5 OCH_3_ protons. ^1^H-NMR spectrum of **4k** revealed signals at 3.74, 3.77, 3.81, 3.91 ppm for 7 OCH_3_ groups. ^13^C-NMR spectrum of **4k** exhibited signals at 55.3, 56.8, 62.7 ppm due to 7 OCH_3_ protons. IR spectra of compounds **4l**–**n** showed bands of NO_2_ groups at their specified regions. ^1^H-NMR spectrum of **4l** revealed singlet at 2.1 ppm and attributed to the 2CH_3_ at the *ortho* positions. ^13^C-NMR spectrum of **4l** exhibited signals at 18.0 ppm for 2CH_3_ protons. ^1^H-NMR spectrum of **4m** revealed singlet at 2.2 ppm attributed to the 2CH_3_ at the *ortho* positions. ^13^C-NMR spectrum of **4m** exhibited signals at 18.0 ppm for 2CH_3_ protons. ^1^H-NMR spectrum of **4n** exhibited singlet at 3.8 ppm attributed to the OCH_3_ moiety, 3.9, 4.2 ppm for 2CH_2_ protons. ^13^C-NMR spectrum of **4n** showed signals at 28.6, 40.4 ppm assigned to 2CH_2_ protons, 57.3 ppm for OCH_3_ protons. 

### 2.2. MTT Antiproliferative Evaluation

Compounds **4a**–**n** were subjected to the cytotoxicity screening of the cell lines MCF-7 (breast), LoVo (colon), HepG2 (liver) and A549 (lung) carcinoma cells as well as HUVEC normal cell lines. The MTT assay was employed to assess cytotoxicity after 48 h of treatment with different concentrations of each compound and doxorubicin was used as a reference drug. The results were presented in Table 1 as growth inhibitory concentration (IC_50_ values). Our results demonstrate that six compounds (**4a**, **4b**, **4d**, **4f**, **4h** and **4i**) out of fourteen exhibited promising antiproliferative activities. A remarkable antiproliferative activity was observed for compound **4d** with an IC_50_ values of 2.5, 5.6, 6.87 and 9 µM against MCF-7, A549, LoVo and HepG2 cells, respectively. Additionally, compound **4f** revealed a promising activity against all tested cell lines with an IC_50_ value of 5, 9.76, 10.14 and 11.7 µM against MCF-7, LoVo, A549 and HepG2 cells, respectively. Interestingly, compound **4f** had a lower cytotoxic effect in terms of IC_50_ values against normal HUVEC cells (IC_50_ = 17.9). Structure activity relationship revealed that 4-tolyl **4d** and 3-ethylphenyl **4f** (IC_50_ value 2.5 and 5.0 µM) showed a demonstrated superior cytotoxic activity and are nearly as active as a reference drug doxorubicin (IC_50_ value 1.4 µM) against MCF-7 breast cancer cell line. It is obvious that replacement of 4-tolyl moiety **4d** (IC_50_ value 2.5 µM) with 2-tolyl fragment **4b** (IC_50_ value 36.2 µM) led to increase the antiproliferative activity against MCF-7 cell line, while incorporation of unsubstituted phenyl moiety as in compound **4a** (IC_50_ value 14.2 µM) with a 4-tolyl fragment **4d** (IC_50_ value 2.5 µM) exhibited intermediate activity. On the contrary, insertion of a methyl group at 3-position **4c** abolishes the cytotoxicity, as opposed to compound **4d** (IC_50_ value 2.5 µM) containing the methyl group in 4-position. On the other hand, the orientation of ethyl group significantly affected the cytotoxic activity of compounds **4e**, **4f**, and **4g** where the presence of it at 3-position as in compound **4f** enhanced the cytotoxic activity (IC_50_ value 5.0 µM) compared to compounds **4e** and **4g** carrying the ethyl group at 2-position and 4-position, respectively, which assigned no antiproliferative completely. Additionally, we observed that **4d** and 3-ethylphenyl moiety **4f** had a considerable potent cytotoxic activity compared to the corresponding unsubstituted phenyl **4a** (IC_50_ value 14.2 µM) and 2-tolyl **4b** (IC_50_ value 36.2 µM). Based on this result, compounds **4d** and **4f** were selected for further investigations. 

### 2.3. Compounds ***4d*** and ***4f*** Induces G1-Phase Cell Cycle Arrest on MCF-7 Cells

**Figure 2:**
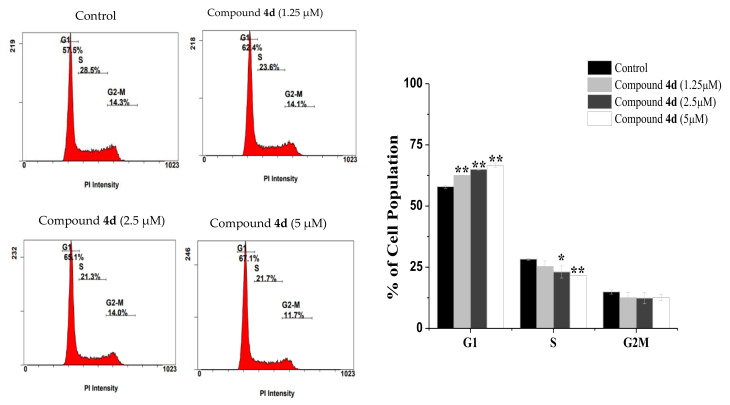
The effects of compound **4d** on cell cycle distribution. MCF-7 cells untreated (control) or treated with the indicated concentrations of compound **4d** for 24 h followed by staining with PI dye. Representative histograms (upper) and graph bar (down) showed the variation in the percentage of cells in each phase of the cell cycle. Results are presented as the mean ± SD. Differences between treated and control groups were considered statistically significant at *p* < 0.05 (*) and *p* < 0.01 (**).

**Figure 3:**
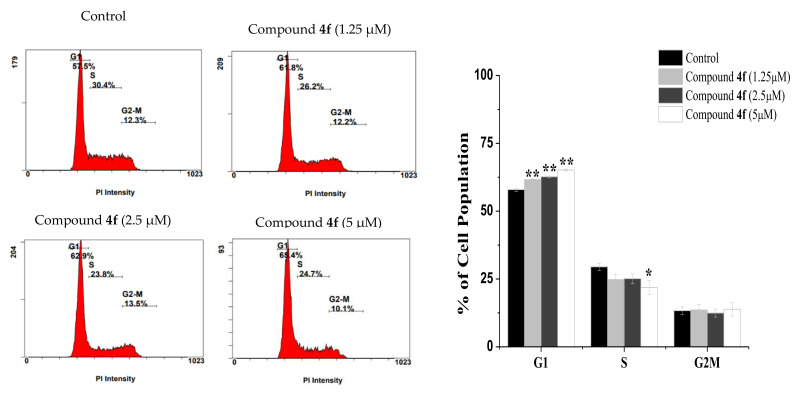
Cell cycle distribution following 24 h exposure to different concentration of compound **4f**. A statistically significant increase in the number of cells in G1 phase was observed in treated cells when compared to control cells. * *p* < 0.05, ** *p* < 0.01.

Because compounds **4d** and **4f** displayed the highest activity towards MCF-7 cells, we looked into their cellular effects with respect to cell cycle progression and cell death mode. Indeed, designing of new chemotherapeutic drugs targeting cell cycle progression and apoptosis is considered as an attractive strategy [23,24]. To explore the antiproliferative effect of compounds **4d** and **4f**, cell-cycle analysis was performed in MCF-7 cells were examined. The cells were treated with various concentrations of both compound for 24 h, stained with propidium iodide (PI) and analyzed by flow cytometry. As shown in (Figure 2), the incubation of MCF-7 cells with (1.25, 2.5, and 5 μM) of compound **4d** for 24 h increased the percentage of cells at G1 (from 57.91 ± 0.61% in control) to 62.51 ± 0.14%, 64.95 ± 0.21% and 66.55 ± 0.78% in treated cells, respectively. At the same conditions, the percentage of cells in S and G2/M phases were obviously decreased. These results revealed that compound **4d** arrested the cell cycle at the G1 stage. In the same manner, DNA content analyses also showed a statistically significant increase in the number of cells in the G1 phase of MCF-7 cells treated with compound **4f** (increased to 61.65 ± 0.21% at 1.25 μM, 62.65 ± 0.35% at 2.5 μM and 65.15 ± 0.36% at 5 μM) when compared to control (57.9 ± 0.61%) (Figure 3). Collectivity, our data indicated that both compounds **4d** and **4f** inhibit MCF-7 cells proliferation by modulating cell cycle progress at G1 phase which is a critical stage in the cell cycle as well as a worth target for drug action [25].

### 2.4. Apoptosis Quantification

**Figure 4:**
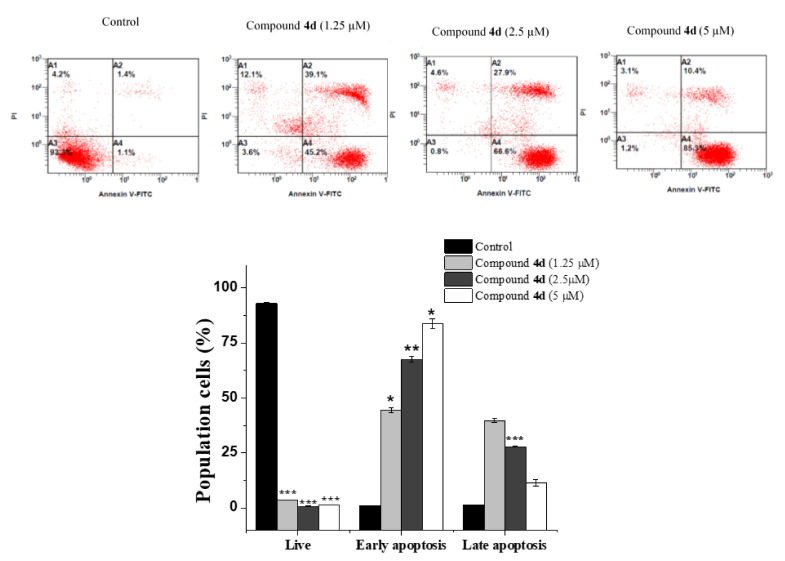
Annexin V-FITC/PI data of untreated and treated MC-7 cells with compound **4d** at the indicated concentrations for 24 h. Flow cytometric plot: A3 (live), A4 (early apoptosis), A2 (late apoptosis) and A1 (necrosis). The bar graph depicts the variation in the percentage of cells in different stages. Results are presented as the mean ± SD and differences between treated and control groups were considered statistically significant at * *p* < 0.05, ** *p* < 0.01, *** *p* < 0.001).

**Figure 5:**
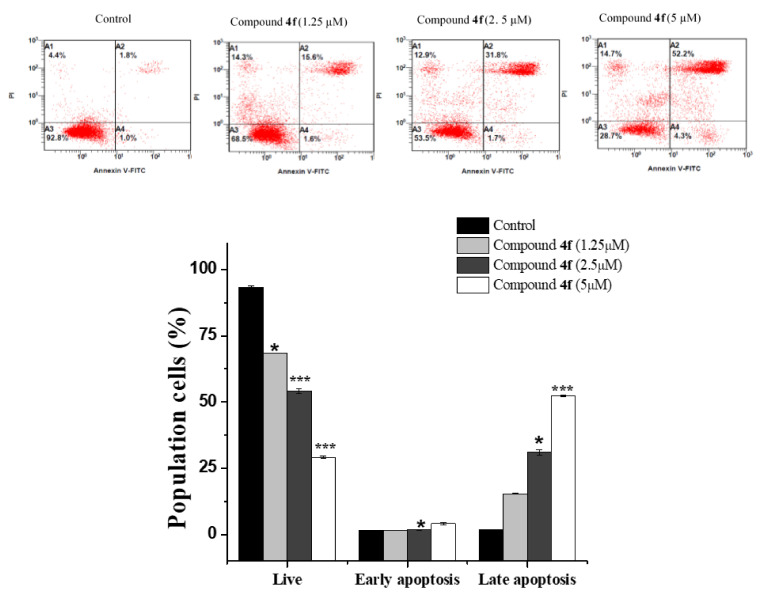
Flow cytometry analysis of compound **4f** for MCF-7 cells following 24 h incubation period at indicated concentrations. Dot plot of Annexin V-FITC/PI and bar quantification for evaluation of apoptosis. Values are presented as means ± SD. Significant differences from control group: * *p* < 0.05, and *** *p* < 0.001.

In addition to cell cycle arrest, apoptosis is another important anticancer process that inhibits cancer cell proliferation [26]. Therefore, we further used a double labeling staining with Annexin V-FITC and PI to investigate apoptotic cell death. Phosphatidylserine exposure on the cell surface is a hallmark of early and late apoptotic cells, which can be easily identified with Annexin V-FITC [27]. As shown in (Figure 4), early apoptotic cells were increased along with the concentrations increment of compound **4d** from 1.05 ± 0.07% in untreated MCF-7 cells to 44.45 ± 1.06% at 1.25 µM, (*p* < 0.05), 67.55 ± 1.34% at 2.5 µM (*p* < 0.01), and 83.75 ± 2.19% at 5 µM (*p* < 0.001). For compound **4f** (Figure 5), it was noticed that most treated cells were in a late apoptotic stage which were increased in a dose-dependent manner (from 1.75 ± 0.07% in control to 15.4 ± 0.28%, 31.05 ± 1.06% and 52.4 ± 0.28% at 1.25, 2.5 and 10 µM, respectively). Interestingly, we noticed that most treated cells were in early apoptotic stage after **4d** compound treatment while most cells populations were in a late apoptotic stage with compound **4f** and these different effects of both compounds could be due to their difference attached functional groups (4-tolyl in **4d** and 3-ethylphenyl in **4f**) which may be targeting different sites for inducing apoptosis in different time periods which need further investigation. Overall, the findings obviously showed that both compounds effectively triggered apoptosis in MCF-7 cells.

### 2.5. Effect of Compounds ***4d*** and ***4f*** on Apoptosis Regulatory Markers 

**Figure 6:**
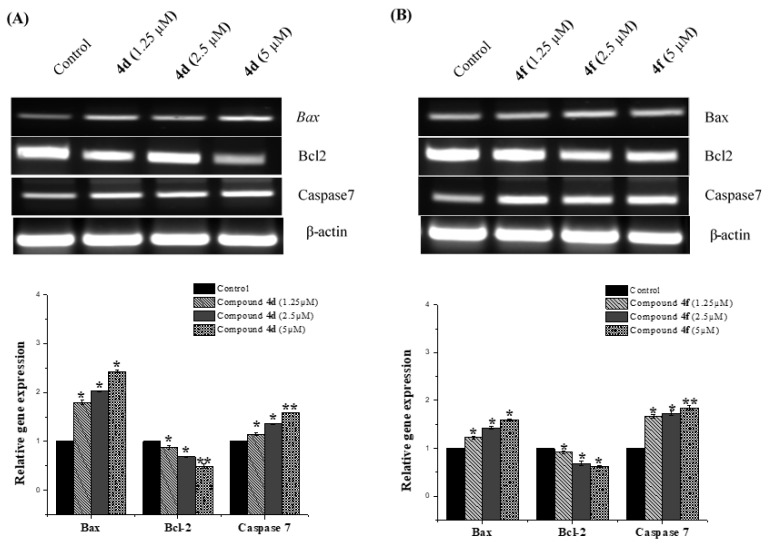
RT-PCR analysis of apoptotic related genes. The RT-PCR expression profile of Bax, Bcl2 and caspase 7 genes were demonstrated in MCF-7 cells after treating them without and with different concentration of compounds **4d** (**A**) and **4f** (**B**) for 24 h. A dose dependent increase in the expression of Bax and caspase 7 with the increasing concentration of the compounds **4d** and **4f** (1.25, 2.5 and 5 µM) was documented while Bcl-2 was downregulated as compared to that of the control where the cells untreated. The levels of mRNA, normalized to that of the β-actin gene, were calculated (histograms). The results were expressed as the mean ± SD. * *p* < 0.05; ** *p* < 0.01 vs. normal control group.

**Figure 7:**
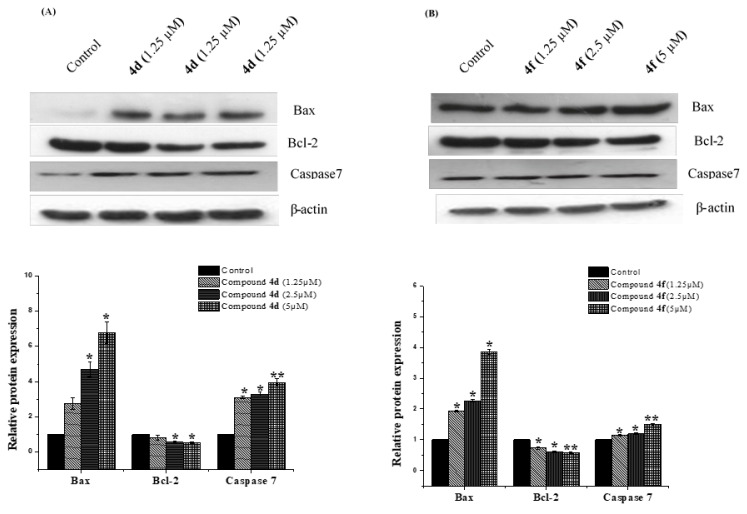
Impact of compounds **4d** and **4f** at various on the expression of apoptosis related genes. MCF-7 cells were treated with vehicle or indicated concentrations (1.25, 2.5 and 5 μM), respectively, for 24 h time period. (**A**) After the treatment, Western blot analysis of Bax, Bcl-2 and caspase 7 was performed. Loading was normalized to the levels of β-actin (**B**) Band quantification of target proteins was performed using Image j software. Data shown are means ± SD (*n* = 3) (* *p* < 0.05, ** *p* < 0.01).

We next used reverse transcription polymerase chain reaction (RT-PCR) and Western blot analysis to look at Bax (proapoptotic), Bcl-2 (antiapoptotic) and caspase 7 (executioner caspase) expression at gene and protein level as an additional marker of apoptosis occurrence [28]. In fact, apoptotic cell death is mostly directed by the levels of Bcl-2 family, whether they are pro-apoptotic or antiapoptotic and targeting these molecules as a therapeutic strategy is continuously under development [29]. As predicted, we observed an upregulation in the expression of Bax in MCF-7 treated cells to (1.7-, 2- and 2.4-fold) with compound **4d** and to (1.2-, 1.4- and 1.6-fold) with compound **4f** at (1.25, 2.5 and 5 µM) respectively (Figure 6). In contrast, Bcl-2 expression was downregulated into (0.88-, 0.68- and 0.49-fold) with **4d** and into (0.92, 0.68 and 0.62) with **4f** at indicated concentration in compare to DMSO-vehicle treated cells. Our results also showed that both **4d** and **4f** caused a significant increase in the expression of caspase 7 to (1.13-, 1.35- and 1.58-fold) relative to untreated MCF-7 cells (Figure 6). Consistently, Western blot analysis revealed that compounds **4d** and **4f** treatment markedly increased the expression of Bax whereas Bcl-2 was downregulated in a dose-dependent manner compared to that of control cells (Figure 7). This shifting in Bax to Bcl-2 ratio usually started caspases activation which plays a critical role in apoptosis execution [26,30]. Among all caspases, caspase 7 is considered as one of the most crucial executioner caspases that mediate cell death, and its expression strongly correlates with apoptosis stimulation [31,32]. Hence, we evaluated whether compounds **4d** and **4f** (1.25, 2.5 and 5 μM) promote the caspase 7 expression at protein level. We found that caspase 7 protein expression significantly increased after cell treatment with compound **4d** (3-, 3.3- and 3.9-fold) and (1.14-, 1.2- and 1.5-fold) with compound **4f** in comparison to the control, which suggested involvement of caspase 7 activation during compounds treatment. In line with our findings, several studies have demonstrated the apoptotic behavior of quinazoline derivatives through upregulating the proapoptotic genes and downregulating the antiapoptotic genes [33,34,35]. In particular, quinazoline-based anticancer drugs such as lapatinib and gefitinib were found to inhibit cancer cells growth through increasing the Bax and caspases gene levels and downregulating Bcl-2 levels, which is consistent with our finding in this study [36,37].

### 2.6. Molecular Docking

Overexpression of anti-apoptotic Bcl-2 protein has been linked to cancer progression and cell death avoidance. Hence, developing novel Bcl2 inhibitors is considered a promising field in designing chemotherapeutic agents [38]. The molecular docking procedure executed in this study was authenticated as reported by [39]. The X-ray bound ligand, i.e., 1-(2-{[(3S)-3-(aminomethyl)-3,4-dihydroisoquinolin-2(1H)-yl]carbonyl}phenyl)-4-chloro-5-methyl-N,N-diphenyl-1H-pyrazole-3-carboxamide (DRO), was extracted from the crystal structure and re-docked to Bcl-2. The poses of the bound and re-docked ligand were compared, and the root mean square deviation (RMSD) was calculated. The RMSD of the re-docked DRO was found to be 0.3842 Å (Figure S1). Because the calculated RMSD was within the acceptable limit (2.0 Å), we were confident in adopting the docking protocol to predict the binding of compounds **4d** and **4f** with Bcl-2. 

**Figure 8:**
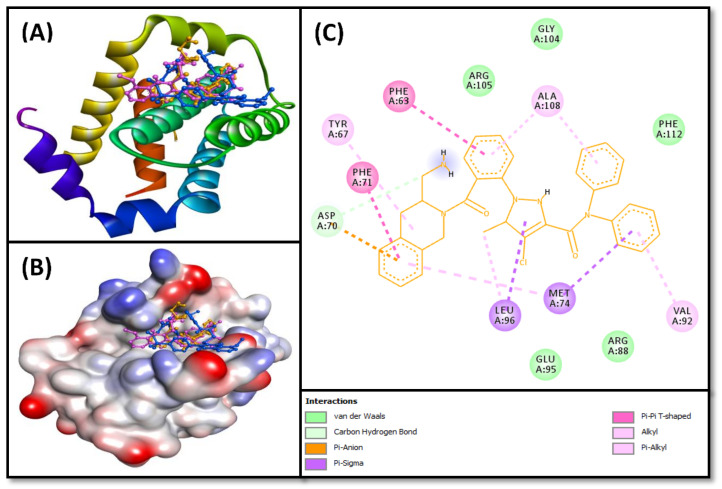
(**A**) 2D cartoon representation of the binding of DRO (control ligand), compounds **4d** and **4f** at the binding site of the Bcl-2 protein, and (**B**) 3D representation of the binding of DRO (control ligand), compounds **4d** and **4f** at the binding site of the Bcl-2 protein. DRO, compounds **4d** and **4f** are represented by gold, pink, and blue, respectively. (**C**) Molecular docking and interaction between Bcl-2 protein and DRO (control ligand).

**Figure 9:**
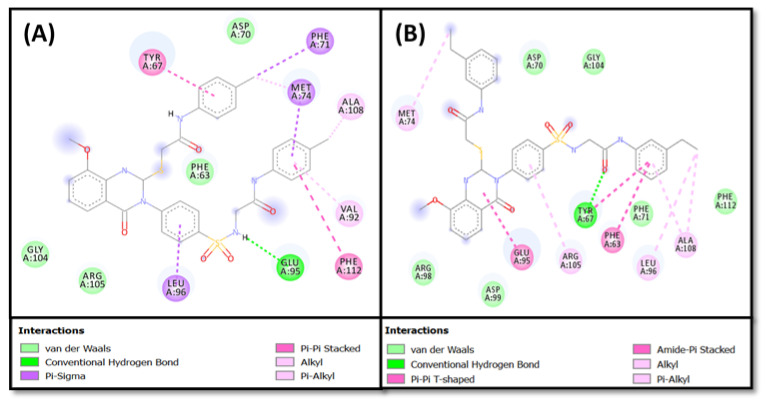
Molecular docking and interaction of (**A**) compound **4d**, and (**B**) compound **4f**, with the Bcl-2 protein.

The results of molecular docking between ligands **4d** and **4f** and Bcl-2 were compared with that of DRO, and the results are given in (Table 2, Figure 8 and Figure 9). We found that both compounds **4d** and **4f** occupied a similar position at the Bcl-2 binding site as occupied by control ligand DRO (Figure 8A,B). The DRO-Bcl-2 complex was stabilized by a carbon interaction and an electrostatic interaction with the Asp70 residue (Figure 8C), as well as ten hydrophobic interactions with Phe63, Tyr67, Phe71, Met74, Val92, Leu96, and Ala108 (Table 2). The DRO-Bcl-2 complex was further stabilized by van der Waals’ interactions of DRO with the Arg88, Glu95, Gly104, Arg105, and Phe112 residues of Bcl-2. Analysis of the interactions between compound **4d** and Bcl-2 revealed that the protein–ligand complex was stabilized by one conventional hydrogen bond with Glu95, and seven hydrophobic interactions with Tyr67, Phe71, Met74, Val92, Leu96, and Ala108 (Table 2). The compound **4d**-Bcl-2 complex was further stabilized by van der Waals’ interactions of the compound with several other residues, including Phe63, Asp70, Gly104, and Arg105 (Figure 9A). Similarly, the compound **4f**-Bcl-2 complex was stabilized by one conventional hydrogen bond with Tyr67, and eight hydrophobic interactions with Phe63, Tyr67, Met74, Glu95, Leu96, Arg105 and Ala108 (Table 2). Additionally, the Asp70, Phe71, Arg98, Asp99, Gly104, and Phe112 residues of Bcl-2 formed van der Waals’ interactions to stabilize the compound **4f**-Bcl-2 complex (Figure 9B). In comparison, the binding energies and the corresponding binding affinities of DRO, compounds **4d** and **4f** towards Bcl-2, were estimated to be −10.2 kcal mol^−1^ and 3.03 × 10^7^ M^−1^, −7.8 kcal mol^−1^ and 5.25 × 10^5^ M^−1^, and −7.5 kcal mol^−1^ and 3.17 × 10^5^ M^−1^, respectively (Table 1). It is interesting to note that Phe63, Tyr67, Asp70, Phe71, Met74, Val92, Glu95, Leu96, Gly104, Arg105, Ala108, and Phe112 residues of Bcl-2 showed interactions with both compounds **4d** and DRO. Similarly, Phe63, Tyr67, Asp70, Phe71, Met74, Glu95, Leu96, Gly104, Arg105, Ala108, Phe112 residues of Bcl-2 showed interactions with compound **4f** as well as the control ligand DRO.

## 3. Experimental

### 3.1. Chemistry

Melting points (uncorrected) were determined in open capillary on a Gallen Kamp melting point apparatus (Sanyo Gallen Kamp, Southborough, UK). Precoated silica gel plates (*Kieselgel* 0.25 mm, 60 F254, Merck, Darmstadt, Germany) were used for thin-layer chromatography. A developing solvent system of chloroform/methanol (8:2) was used and the spots were detected by ultraviolet light. IR spectra (KBr disc) were recorded using an FT-IR spectrophotometer (Perkin Elmer, Norwalk, CT, USA). ^1^H-NMR spectra were scanned on an NMR spectrophotometer (Bruker AXS Inc., Flawil, Switzerland), operating at 500 MHz for ^1^H- and 125.76 MHz for ^13^C. Chemical shifts are expressed in δ-values (ppm) relative to TMS as an internal standard, using DMSO-*d_6_* as a solvent. Elemental analyses were done on a model 2400 CHNSO analyser (Perkin Elmer, Norwalk, CT, USA). All the values were within ±0.4% of the theoretical values. All reagents used were of AR grads.

**4-(2-Mercapto-8-methoxy-4- oxoquinazolin-3(4*H*)-yl) benzenesulfonamide (3)**

A mixture of 2-amino-3-methoxybenzoic acid **1** (1.67 g, 0.01 mol) and 4-isothiocyanatobenzenesulfonamide **2** (2.14 g, 0.01 mol) in dioxan (50 mL) containing 3 drops of triethylamine, was refluxed for 8 h. The solid product formed while hot was collected by filtration and recrystallized from ethanol to give **3**. **3:** Yield, 89%; m.p. 342–344 °C. IR: 3321, 3262, 3181 (NH_2_), 3055 (arom.), 1691 (CO), 1620 (CN), 1381, 1156 (SO_2_). ^1^HNMR: 3.9 (s, 3H, OCH_3_), 7.0–7.7 [m, 5H, Ar-H + SO_2_NH_2_], 7.8, 8.0 (2d. 4H, AB system, Ar-H), 12.3 [s, 1H, SH]. ^13^CNMR: 55.7, 116.2, 118.6, 119.8, 120.6 (2), 127.0, 128.9 (2), 130.1, 134.6, 135.3, 150.4, 160.5, 160.9. MS *m*/*z* (%): 363 (M^+^) (14.54), 155 (100). Anal. Calcd. for C_15_H_13_N_3_O_4_S_2_ (363.41): C, 49.57; H, 3.61; N, 11.56. Found: C, 49.22; H, 3.86; N, 11.31. **Quinazolin-3(4*H*)-yl)phenylsulfonamido)-*N*-phenylacetamide derivatives (4a-n).**

### 3.2. General Procedure

#### 3.2.1. Method A

A mixture of **3** (4.59 g, 0.01 mol) and 2-chloro-*N*-substitutedacetamide derivatives (0.02 mol) in dry acetone (50 mL) and anhydrous K_2_CO_3_ (2.56 g, 0.02 mol) was stirred at room temperature for 24 h., filtered and the solid product formed was recrystallized from ethanol to give **4a**–**n**.

#### 3.2.2. Method B

A mixture of **5a**–**n** [20] (0.01 mol) and 2-chloro-*N*-substitutedacetamide derivatives (0.01 mol) in dry acetone (50 mL) and anhydrous K_2_CO_3_ (1. 38 g, 0.01 mol) was stirred at room temperature for 18 h, filtered and the solid product formed was recrystallized from ethanol to give **4a**–**n**.

**2-(4-(8-Methoxy-4-oxo-2-((2-oxo-2-(phenylamino)ethylthio)quinazolin-3(4H)-yl)phenylsul-fonamido)-N-phenylacetamide (4a).**

**5:** Yield, 90%; m.p. 165–167 °C. IR: 3300, 3141 (NH), 3066 (arom.), 2965, 2844 (aliph.), 1700, 1693, 1665 (3CO), 1612 (CN), 1377, 1154 (SO_2_). ^1^HNMR: 3.8 [s, 3H, OCH_3_], 4.1 [s, 2H, SCH_2_], 4.2 [s, 2H, NCH_2_], 7.5, 7.6 (2d, 4H, AB system, Ar-H), 7.0- 7.3 [m, 13H, Ar-H), 8.0 (s, 1H, SO_2_NH], 10.3 [s, 2H, 2NH]. ^13^CNMR: 31.6, 44.0, 56.7, 116.2, 116.8, 119.8 (4), 120.6, 124.3 (2), 129.3 (2), 129.4 (4), 129.8 (2), 131.7 (2), 137.0, 138.9 (2), 145.7, 154.0, 158.8, 161.4, 165.0, 165.6. MS *m*/*z* (%): 629 (M^+^) (31.43), 231 (100). Anal. Calcd. for C_31_H_27_N_5_O_6_S_2_ (629.71): C, 59.13; H, 4.32; N, 11.12. Found: C, 59.45; H, 4.65; N, 10.83.

**2-(4-(8-Methoxy-4-oxo-2-((2-oxo-2-(*o*-tolylamino)ethylthio)quinazolin-3(4*H*)-yl)phenylsul-fonamido)-*N*-(*o*-tolyl)acetamide (4b).**

**4b:** Yield, 90%; m.p. 102–104 °C. IR: 3354, 3154 (NH), 3036 (arom.), 2972, 2873 (aliph.), 1698, 1668, 1660 (3CO), 1610 (CN), 1386, 1166 (SO_2_). ^1^HNMR: 2.1 [s, 3H, CH_3_], 3.8 [s, 3H, OCH_3_], 4.1 [s, 2H, SCH_2_], 4.3 [s, 2H, NCH_2_], 7.1, 7.7 [m, 11H, Ar-H), 7.9, 8.0 (2d, 4H, AB system, Ar-H), 8.1 (s, 1H, SO_2_NH], 9.6 [s, 2H, 2NH]. ^13^CNMR: 18.1 (2), 31.6, 43.6, 57.8, 116.6, 117.8, 121.0, 124.8 (2), 125.6 (2), 126.1 (2), 126.5, 129.6 (2), 130.8 (2), 132.4 (2), 132.6 (2), 136.0, 137.9, 138.2 (2), 139.8, 147.6, 155.6, 161.2, 165.6, 165.9. MS *m*/*z* (%): 657 (M^+^) (11.84), 188 (100). Anal. Calcd. for C_33_H_31_N_5_O_6_S_2_ (657.76): C, 60.26; H, 4.75; N, 10.65. Found: C, 60.60; H, 4.39; N, 10.36.

**2-(4-(8-Methoxy-4-oxo-2-((2-oxo-2-(*m*-tolylamino)ethylthio)quinazolin-3(4*H*)-yl)phenylsul-fonamido)-*N*-(*m*-tolyl)acetamide (4c).**

**4c:** Yield, 84%; m.p. 112–114 °C. IR: 3309, 3144 (NH), 3078 (arom.), 2954, 2863 (aliph.), 1692, 1677, 1661 (3CO), 1618 (CN), 1377, 1151 (SO_2_). ^1^HNMR: 2.3 [s, 6H, 2CH_3_], 3.9 [s, 3H, OCH_3_], 4.0 [s, 2H, SCH_2_], 4.2 [s, 2H, NCH_2_], 6.8–7.7 [m, 11H, Ar-H), 7.6, 8.0 (2d, 4H, AB system, Ar-H), 9.6 (s, 1H, SO_2_NH], 10.1 [s, 2H, 2NH]. ^13^CNMR: 21.6 (2), 26.7, 40.3, 56.7, 116.8, 117.1 (2), 117.4, 120.1 (2), 120.7, 124.5 (2), 124.8 (2), 127.4, 128.9 (2), 129.0 (2), 130.7, 138.2, 138.3 (2), 138.7 (2), 141.3, 155.6, 161.2, 165.0, 168.5, 171.3. MS *m*/*z* (%): 657 (M^+^) (3.76), 154 (100). Anal. Calcd. for C_33_H_31_N_5_O_6_S_2_ (657.76): C, 60.26; H, 4.75; N, 10.65. Found: C, 60.01; H, 4.43; N, 10.89.

**2-(4-(8-Methoxy-4-oxo-2-((2-oxo-2-(*p*-tolylamino)ethylthio)quinazolin-3(4*H*)-yl)phenylsul-fonamido)-*N*-(*p*-tolyl)acetamide (4d).**

**4d:** Yield, 84%; m.p. 160–162 °C. IR: 3353, 3132 (NH), 3064 (arom.), 2976, 2845 (aliph.), 1695, 1676, 1660 (3CO), 1620 (CN), 1387, 1155 (SO_2_). ^1^HNMR: 2.2 [s, 6H, 2CH_3_], 3.8 [s, 3H, OCH_3_], 4.0 [s, 2H, SCH_2_], 4.2 [s, 2H, NCH_2_], 7.1–7.4 [m, 3H, Ar-H), 7.6, 8.0 (2d, 12H, AB system, Ar-H), 8.1 (s, 1H, SO_2_NH], 10.2 [s, 2H, 2NH]. ^13^CNMR: 20.9 (2), 28.6, 44.0, 56.7, 118.0, 119.8, 120.8 (4), 120.9 (3), 129.6 (5), 129.7 (2), 129.9, 133.2 (2), 136.4, 138.0 (2), 139.2, 153.8, 158.6, 164.8, 165.2 (2). MS *m*/*z* (%): 657 (M^+^) (17.54), 224 (100). Anal. Calcd. for C_33_H_31_N_5_O_6_S_2_ (657.76): C, 60.26; H, 4.75; N, 10.65. Found: C, 60.50; H, 4.55; N, 10.44.

***N*-(2-ethylphenyl)-2-((3-(4-(*N*-(2-((2-ethylphenyl)amino)-2-oxoethyl)sulfamoyl)-phe-nyl)- 8-methoxy-4-oxo-3,4-dihydroquinazolin-2yl) thio)acetamide (4e).**

**4e:** Yield, 64%; m.p. 246–248 °C. IR: 3409, 3256 (NH), 3075 (arom.), 2944, 2856 (aliph.), 1694, 1683, 1665 (3CO), 1617 (CN), 1398, 1155 (SO_2_). ^1^HNMR: 1.0 (t, 6H, 2 CH_3_ ethyl), 2.5 (q, 4H, 2CH_2_ ethyl), 3.8 [s, 3H, OCH_3_], 3.9 [s, 2H, SCH_2_], 4.1 [s, 2H, NCH_2_], 7.1–7.7 [m, 11H, Ar-H), 7.9, 8.0 (2d, 4H, AB system, Ar-H), 8.1 (s, 1H, SO_2_NH], 9.6 [s, 2H, 2NH]. ^13^CNMR: 14.5 (2), 24.0 (2), 31.0, 40.3, 56.7, 116.3, 118.1, 120.9, 126.3 (2), 126.4 (2), 127.1 (2), 127.4 (2), 128.9 (2), 130.3 (2), 130.7 (2), 135.8 (2), 138.0 (2), 139.1 (2), 139.2, 153.7, 161.0, 166.4 (2). MS *m*/*z* (%): 685 (M^+^) (23.62), 288 (100). Anal. Calcd. for C_35_H_35_N_5_O_6_S_2_ (685.81): C, 61.30; H, 5.14; N, 10.21. Found: C, 61.00; H, 5.37; N, 10.46.

***N*-(3-ethylphenyl)-2-((3-(4-(*N*-(2-((3-ethylphenyl)amino)-2-oxoethyl)sulfamoyl)-phe-nyl)-8-methoxy-4-oxo-3,4-dihydroquinazolin-2yl) thio)acetamide (4f).**

**4f:** Yield, 57%; m.p. 239–241 °C. IR: 3321, 3123 (NH), 3084 (arom.), 2975, 2866 (aliph.), 1691, 1685, 1660 (3CO), 1619 (CN), 1376, 1151 (SO_2_). ^1^HNMR: 1.2 (t, 6H, 2CH_3_ ethyl), 2.6 (q, 4H, 2CH_2_), 3.8 [s, 3H, OCH_3_], 4,0 [s, 2H, SCH_2_], 4.2 [s, 2H, NCH_2_], 6.9–7.6 [m, 11H, Ar-H), 7.6, 8.0 (2d, 4H, AB system, Ar-H), 9.5 (s, 1H, SO_2_NH], 10,4 [s, 2H, 2NH]. ^13^CNMR: 15.9 (2), 28.6 (2), 28.7, 40.3, 56.7, 116.4, 117.0 (2), 117.2, 118.1 (2), 119.1, 120.9 (2), 123.8 (2), 127.0, 127.4 (2), 129.2 (2), 130.7, 138.0, 139.3 (2), 144.7, 145.9 (2), 153.7, 155.6, 160.9, 165.0, 166.0. MS *m*/*z* (%): 685 (M^+^) (10.74), 412 (100). Anal. Calcd. for C_35_H_35_N_5_O_6_S_2_ (685.81): C, 61.30; H, 5.14; N, 10.21. Found: C, 61.03; H, 4.87; N, 10.06.

***N*-(4-ethylphenyl)-2-((3-(4-(*N*-(2-((4-ethylphenyl)amino)-2-oxoethyl)sulfamoyl)-phenyl)-8-methoxy-4-oxo-3,4-dihydroquinazolin-2yl)thio)acetamide (4g).**

**4g:** Yield, 64%; m.p. 219–221 °C. IR: 3409, 3256 (NH), 3075 (arom.), 2944, 2856 (aliph.), 1694, 1683, 1665 (3CO), 1617 (CN), 1398, 1155 (SO_2_). ^1^HNMR: 1.1 (t, 6H, 2CH_3_ ethyl), 2.5 (q, 4H, 2CH_2_ ethyl_)_, 3.8 [s, 3H, OCH_3_], 4.0 [s, 2H, SCH_2_], 4.2 [s, 2H, NCH_2_], 7.1–7.5 [m, 3H, Ar-H), 7.6, 8.0 (2d, 12H, AB system, Ar-H), 8.1 (s, 1H, SO_2_NH], 10.2 [s, 2H, 2NH]. ^13^CNMR: 16.1 (2, 28.0 (3), 44.0, 58.6, 115.6, 117.8, 119.9 (4), 120.1, 121.6 (2), 126.7 (4), 127.8, 128.5 (2), 129.8, 136.6 (2), 136.8, 139.7, 140.2 (2), 153.9, 161.2, 164.8, 165.6 (2). MS *m*/*z* (%): 685 (M^+^) (23.62), 288 (100). Anal. Calcd. for C_35_H_35_N_5_O_6_S_2_ (685.81): C, 61.30; H, 5.14; N, 10.21. Found: C, 61.66; H, 5.39; N, 10.04.

**2-(4-(8-Methoxy-2-((2-((4-methoxyphenyl)amino)-2-oxoethyl)thio)-4-oxoquinazolin-3(4*H*)-yl)phenylsulfonamido)-*N*-(4-methoxy-phenyl)acetamide (4h).**

**4h:** Yield, 71%; m.p. 117–119 °C. IR: 3350, 3231 (NH), 3054 (arom.), 2986, 2891 (aliph.), 1703, 1692, 1664 (3CO), 1611 (CN), 1364, 1152 (SO_2_). ^1^HNMR: 3.72, 3.73, 3.88 [3s, 9H, 3OCH_3_], 4.1 [s, 2H, SCH_2_], 4.2 [s, 2H, NCH_2_], 6.9, 7.5 (2d, 8H, AB system, Ar-H), 7.5–7.6 (m, 3H, Ar-H), 7.6, 8.0 (2d, 4H, AB system, Ar-H), 8.1 (s, 1H, SO_2_NH], 10.5 [s, 2H, 2NH]. ^13^CNMR: 31.3, 43.9, 55.6 (3), 114.4 (4), 116.8, 118.6, 121.4, 123.7 (2), 125.8 (4), 125.9, 128.6 (2), 129.1 (2), 132.0, 137.2, 137.6, 155.9, 156.0 (2), 162.6, 164.6, 165.2 (2). MS *m*/*z* (%): 689 (M^+^) (9.86), 312 (100). Anal. Calcd. for C_33_H_31_N_5_O_8_S_2_ (689.76): C, 57.46; H, 4.53; N, 10.15. Found: C, 57.13; H, 4.23; N, 10.35.

***N*-(4-Ethoxyphenyl)-2-((3-(4-*N*-(2((4-ethoxyphenyl)amino)-2-oxoethyl)sulfamoyl)-phenyl-8-methoxy-4-oxo-3,4-dihydroquinazolin-2-yl)thio)acetamide (4i).**

**4i:** Yield, 66%; m.p. > 350 °C. IR: 3343, 3222 (NH), 3077 (arom.), 2965, 2836 (aliph.), 1698, 1685, 1666 (3CO), 1611 (CN), 1367, 1155 (SO_2_). ^1^HNMR: 1.3 (t, 6H, 2CH_3_ ethyl), 3.9 [s, 3H, OCH_3_], 4.0 (q, 4H, 2CH_2_ ethyl), 4.1 [s, 2H, SCH_2_], 4.2 [s, 2H, NCH_2_], 6.8, 7.4 (2d, 8H, AB system, Ar-H), 7.6–7.8 (m, 3H, Ar-H), 7.9, 8.0 (2d, 4H, AB system, Ar-H), 8.1 (s, 1H, SO_2_NH], 10.1 [s, 2H, 2NH]. ^13^CNMR: 15.1 (2), 31.3, 43.9, 57.2, 63.5 (2), 114.9 (4), 116.0, 118.6, 121.3, 121.4 (2), 126.2 (4), 126.3, 129.7 (2), 131.9 (2), 132.0, 138.9, 139.0, 155.1, 157.4 (2), 161.6, 164.5, 165.1 (2). MS *m*/*z* (%): 717 (M^+^) (51.82), 365 (100). Anal. Calcd. for C_35_H_35_N_5_O_8_S_2_ (717.81): C, 58.56; H, 4.91; N, 9.76. Found: C, 58.21; H, 4.65; N, 9.45.

***N*-(3,5-Dimethoxyphenyl)-2-((3-(4-*N*-(2((3,5-dimethoxyphenyl)amino)-2-oxoethyl)-sulfa-moyl)phenyl-8-methoxy-4-oxo-3,4-dihydroquinazolin-2-yl)thio)acetamide (4j).**

**4j:** Yield, 65%; m.p. 239–241 °C. IR: 3384, 3198 (NH), 3045 (arom.), 2978, 2865 (aliph.), 1700, 1681, 1662 (3CO), 1612 (CN), 1356, 1154 (SO_2_). ^1^HNMR: 3.81, 3.84, 3.90 [3s, 15H, 5OCH_3_], 4.0 [s, 2H, SCH_2_], 4.1 [s, 2H, NCH_2_], 6.2–7.6 [m, 9H, Ar-H), 7.8, 8.0 (2d, 4H, AB system, Ar-H), 8.2 (s, 1H, SO_2_NH], 10.1 [s, 2H, 2NH]. ^13^CNMR: 27.6, 41.8, 56.3 (5), 97.2 (2), 101.0 (4), 114.0, 117.2, 120.3, 120.9 (2), 127.4, 128.3 (2), 131.9, 134.6, 139.7, 140.6 (2), 151.8, 158.6, 159.0 (4), 161.2, 167.4, 168.2, MS *m*/*z* (%): 749 (M^+^) (1.85), 147 (100). Anal. Calcd. for C_35_H_35_N_5_O_10_S_2_ (749.81): C, 56.06; H, 4.70; N, 9.34. Found: C, 56.29; H, 4.45; N, 9.66.

**2-(4-(8-Methoxy-4-oxo-2-((2-oxo-2-(3,4,5 trimethoxyphenyl)amino)ethyl)thio)quinazolin-3(4*H*)-yl)phenylsulfonamido)-*N*-(3,4,5-trimethoxyphenyl)acetamide (4k).**

**4k:** Yield, 78%; m.p. 260–262 °C. IR: 3377, 3154 (NH), 3043 (arom.), 2933, 2875 (aliph.), 1690, 1672, 1665 (3CO), 1623 (CN), 1376, 1151 (SO_2_). ^1^HNMR: 3.74, 3.77, 3.81, 3.91 [4s, 21H, 7OCH_3_], 4.0 [s, 2H, SCH_2_], 4.1 [s, 2H, NCH_2_], 6.9–7.7 [m, 7H,), 7.8, 8.0 (2d, 4H, AB system, Ar-H), 8.3 (s, 1H, SO_2_NH], 10.2 [s, 2H, 2NH]. ^13^CNMR: 28.3, 42.7, 55.3, 56.8 (4), 62.7 (2), 97.0 (4), 114.6, 120.6, 120.8, 121.2 (2), 129.8, 130.5 (2), 131.7, 133.0, 134.2, 136.4 (2), 137.1, 139.6, 151.8, 155.1 (4), 158.3, 161.0, 168.1, 168.9. MS *m*/*z* (%): 809 (M^+^) (3.42), 364 (100). Anal. Calcd. for C_37_H_39_N_5_O_12_S_2_ (809.86): C, 54.87; H, 4.85; N, 8.65. Found: C, 54. 49; H, 4.58; N, 8.28.

**2-(4-(8-Methoxy-2-((2-((2-methyl-4-nitrophenyl)amino)-2-oxoethyl)thio)-4-oxoquinazolin-3(4*H*)-yl)phenylsulfonamido)-*N*-(2-methyl-4-nitrophenyl)acetamide (4l).**

**4l:** Yield, 61%; m.p. 127–129 °C. IR: 3325, 3245 (NH), 3076 (arom.), 2976, 2843 (aliph.), 1694, 1682, 1660 (3CO), 1608 (CN), 1387, 1161 (SO_2_). ^1^HNMR: 2.1 (s, 6H, 2CH_3_), 3.7 [s, 3H, OCH_3_], 3.9 [s, 2H, SCH_2_], 4.2 [s, 2H, NCH_2_], 7.4–7.8 (m, 9H, Ar-H), 7.9, 8.0 (2d, 4H, AB system, Ar-H), 8.1 (s, 1H, SO_2_NH], 9.9 [s, 2H, 2NH]. ^13^CNMR: 18.0 (2), 28.6, 40.3, 56.5, 112.2 (2), 116.3, 118.0, 120.9 (2), 122.2, 125.8 (2), 127.1 (2), 127.4 (2), 130.7 (2), 130.9, 138.0, 139.1 (2), 143.2 (2), 145.9 (2), 153.7, 161.0, 166.9 (2), 172.0. MS *m*/*z* (%): 747 (M^+^) (16.66), 384 (100). Anal. Calcd. for C_33_H_29_N_7_O_10_S_2_ (747.75): C, 53.01; H, 3.91; N, 13.11. Found: C, 53.33; H, 3.62; N, 13.43.

**2-(4-(8-Methoxy-2-((2-((2-methyl-6-nitrophenyl)amino)-2-oxoethyl)thio)-4-oxoquinazolin-3(4*H*)-yl)phenylsulfonamido)-*N*-(2-methyl-6-nitrophenyl)acetamide (4m).**

**4m:** Yield, 85%; m.p. 272–274 °C. IR: 3340, 3212 (NH), 3055 (arom.), 2954, 2865 (aliph.), 1695, 1684, 1661 (3CO), 1618 (CN), 1377, 1151 (SO_2_). ^1^HNMR: 2.2 (s, 6H, 2CH_3_), 3.8 [s, 3H, OCH_3_], 3.9 [s, 2H, SCH_2_], 4.1 [s, 2H, NCH_2_], 7.4–7.5 (m, 9H, Ar-H), 7.6, 8.0 (2d, 4H, AB system, Ar-H), 8.1 (s, 1H, SO_2_NH], 10.2 [s, 2H, 2NH]. ^13^CNMR: 18.0 (2), 31.6, 40.4, 56.7, 115.3, 118.1, 120.9, 122.6 (2), 123.0 (2), 126.7 (2), 127.1 (2), 127.4, 127.5 (2), 128.8 (2), 130.3, 135.4 (2), 137.7, 137.9 (2), 139.0, 153.7, 161.0, 162.5, 166.4 (2). MS *m*/*z* (%): 747 (M^+^) (28.14), 341 (100). Anal. Calcd. for C_33_H_29_N_7_O_10_S_2_ (747.75): C, 53.01; H, 3.91; N, 13.11. Found: C, 52.76; H, 4.22; N, 13.35.

***N*-(2,4-dinitrophenyl)-2-((3-(4-*N*-(2((2,4-dinitrophenyl)amino)-2-oxoethyl)sulfamoyl)phen-yl-8-methoxy-4-oxo-3,4-dihydroquinazolin-2-yl)thio)acetamide (4n).**

**4n:** Yield, 76%; m.p. 248–250 °C. IR: 3342, 3200 (NH), 3093 (arom.), 2984, 2876 (aliph.), 1696, 1681, 1669 (3CO), 1608 (CN), 1398, 1166 (SO_2_). ^1^HNMR: 3.8 [s, 3H, OCH_3_], 3.9 [s, 2H, SCH_2_], 4.2 [s, 2H, NCH_2_], 7.1–7.6 (m, 9H, Ar-H), 7.7, 7.9 (2d, 4H, AB system, Ar-H), 8.1 (s, 1H, SO_2_NH], 8.8 [s, 2H, 2NH]. ^13^CNMR: 28.6, 40.4, 57.3, 115.1, 120.2, 123.9 (2), 124.0, 125.6 (2), 126.9 (2), 129.1, 129.8 (2), 130.6 (2), 135.5, 136.7, 138.0 (2), 138.7, 138.9, 143.1 (2), 147.3, 150.3, 156.4, 161.0, 163.5, 164.6. MS *m*/*z* (%): 809 (M^+^) (16.42), 294 (100). Anal. Calcd. for C_31_H_23_N_9_O_14_S_2_ (809.70): C, 45.98; H, 2.86; N, 15.57. Found: C, 45.64; H, 2.56; N, 15.25.

### 3.3. Biological Evaluations 

#### 3.3.1. MTT Cell Viability Assay

Cell viability was determined using 3-(4,5-dimethylthiazol-2-yl)-2,5-diphenyl tetrazolium bromide (MTT) assay as stated previously [40]. In brief, A549 (lung), HepG2 (liver), LoVo (colon), MCF-7 (breast) cancer cells as well as normal cells (HUVEC) were seeded in 96-well plates (10^5^ per well) and compounds were added in a different final concentration. All cells were treated for 48 h, and then 10μL of MTT reagent (5 mg/mL) was added to each well and further incubated for 4 h. Thereafter, 0.1 mL of acidified isopropanol was added to solubilize formazan product and the developed color was measured at 570 nm in Microplate ELISA Reader (BioTek, Elx-800, Winooski, VT, USA). The IC_50_ values (concentration required for 50% inhibition of cell viability) were calculated using dose response curve. Cell viability was calculated as = (Ab Treated/Ab Control) × 100. 

The A549, HepG2, LoVo and MCF-7 cancer cell lines were obtained from the German Collection of Microorganisms and Cell Cultures (DSMZ) (Braunschweig, Germany). For Human umbilical vein endothelial cells (HUVEC cells), it was purchased from American Type Culture Collection (16549; Cat no. PCS-100-010; ATTCC, Manassas, VA, USA).

#### 3.3.2. Cell Cycle Analysis

Cell cycle distribution was evaluated by PI staining as previously described [41]. Briefly, MCF-7 cells were seeded and incubated for a period of 24 h to allow for attachment. Subsequently, cells were exposed to different concentrations of compounds **4d** and **4f**. After 24 h, cells were harvested and fixed in 70% ethanol. Thereafter, cells were washed with PBS, resuspended in 1 mL of PBS containing PI (50 μg/mL), RNase A (0.5 mg/mL), sodium citrate (0.1%) and Triton X-100 (0.1%), then incubated for 15 min in the dark at room temperature. Flow cytometry was carried out by a FACS Scan Flow Cytometer (Cytomics FC 500; Beckman Coulter, Brea, CA, USA). The data were analyzed using CXP software V. 3.0.

#### 3.3.3. Annexin V-FITC/PI Apoptosis Detection

Apoptotic cells were detected using Annexin V-FITC/PI staining protocol (BioLegend, San Diego, CA, USA) according to the manufacturer’s recommendations. Briefly, MCF-7 cells were incubated for 24 h with the different doses of promising compounds **4d** and **4f**. The cells were then harvested, washed twice with cold PBS and re-suspended with 1× Annexin V binding buffer (100 µL). Thereafter, cells were transferred into flow cytometry tube and stained with 5 μL of Annexin V- FITC and 5 μL PI for 15 min at room temperature in the dark. The cells were processed for apoptosis using a FACS Scan Flow Cytometer (Cytomics FC 500; Beckman Coulter, Brea, CA, USA) and analyzed using CXP software V. 3.0.

#### 3.3.4. Analysis of Apoptotic Genes by RT-PCR

The MCF-7 cells (0.1 × 10^6^) in 12 well plates were treated with different concentrations of compounds **4d** and **4f** for 24 h and untreated cells were used as a control. After 24 h of treatment, cell culture media was removed and the cells were lysed with Trizol (Invitrogen, Waltham, MA, USA), and total RNA was extracted from all the samples [42]. The RNA was estimated by Nano-drop and 1 µg of RNA was used in the cDNA synthesis. The cDNA was synthesized from RNA by using a cDNA synthesis kit (Invitrogen, Thermo Fisher Scientific, lnc., Waltham, MA, USA) according to the manufacturer’s instruction. After Reverse transcription at 42 °C for 60 min, RT-PCR was performed using 5× Firepol Master Mix ready to load (Solis BioDyne) according to manufacturer’s instruction. RT-PCR was used to analyze the gene expression level of *P53*, *Bcl2*, *Bax* and *caspase 7* using specific sets of primers for each gene [22], while β-actin was utilized as a housekeeping gene. The program was run as follows, initial denaturation at 95 °C for 5 min; denaturation at 95 °C for 30 s; annealing at 55 °C for 45 s; elongation at 72 °C for 45 s (30 Cycles) and final extension at 72 °C for 10 min. The RT-PCR products were electrophoresed on a 1.2% agarose gel containing ethidium bromide and the gel was imaged on a Licor machine.

#### 3.3.5. Western Blotting to See the Effect of **4d** and **4f** Compounds

MCF-7 cells were plated in six well plates at the density of 0.5 × 10^6^ and kept for adherence for overnight. On the next day, complete media was replaced with the fresh one and treated the cells with compounds **4d** and **4f** for 24 h at various concentrations. After 24 h of treatment, cells were harvested and washed twice with 1× PBS before adding the lysis buffer (20 mM Tris pH 7.5, 150 mM Nacl, 1 mM Na2EDTA, 1 mM EGTA, 1% Triton X100, 1 μg/mL Leupeptin and 100 µM phenylmethylsulfonyl fluoride—PMSF) and kept it for 1 h on ice, with intermittent vortex mixing every 10 min. The samples were centrifuged at 13,000 rpm at 4 °C for 15 min and then the supernatants were transferred into new tubes. Total protein was measured by using Bio-Rad Bradford kit and then 35 µg of the total protein was run on a 12% SDS-PAGE and transferred to a nitrocellulose membrane. The membrane was blocked with 5% BSA in TBST (150 mM Nacl, 20 mM Tris base and 0.1% Tween 20) for 2 h at room temperature and then washed three times with TBST buffer. Then the membrane was incubated with the desired antibodies (p53, Bcl-2, Bax, caspase7 and β-actin) (1:200, Santa Cruz Biotecnology, Inc., Dallas, TX, USA) and kept on a rocker at 4 °C overnight. On the next day, the membrane was washed three times with TBST buffer and then incubated with horseradish peroxidase-conjugated secondary antibody (1:3000) for 1 h at room temperature. After incubation, membranes were washed three times with TBST buffer and then were ready for immunodetection. For immunodetection, membranes were incubated with ECL Western blotting detecting reagents (Amersaham Pharmacia Biotech Inc., Zurich, Switzerland) and then bands were obtained by exposing to X-ray films (Amersham). 

#### 3.3.6. Molecular Docking

Molecular docking between potential compounds **4d** and **4f** with the target protein Bcl-2 was performed as reported earlier [43]. The X-ray crystal structure Bcl-2 bound with DRO (2.10 Å resolution) was used to retrieve 3D coordinates of the target protein [44]. The protein was processed to remove non-essential water molecules and other heterogeneous atoms. Hydrogen atoms were added, bond orders were defined, and rotatable bonds were assigned, as reported by [45]. Prior to molecular docking, hydrogen bonds were redefined, and the energy of the protein was minimized using MMFF (Merck Molecular Force Field) force field. The 2D structures of compounds **4d** and **4f** were drawn using ChemDraw, and their energies were minimized using Universal Forcefield (UFF). Molecular docking between Bcl-2 and ligands (**4d** and **4f**) along with the control ligand (DRO) was performed inside a grid box of 27 × 30 × 25 Å, placed at 39 × 28 × −12 Å, keeping 0.375 Å spacing between the grid points, using Autodock 4.2 (Scripps Research, San Diego, CA, USA), as reported by Rabbani and coworkers [46]. Lamarckian Genetic Algorithm (LGA) and Solis and Wets local search methods were used to perform molecular docking. Initial torsions, positions, and orientations of ligands were set arbitrarily. A maximum of 2,500,000 calculations was for each docking run. The population size, translational step, quaternion and torsion were set to 150, 0.2 Å, 5 and 5, respectively. The docked protein-ligand complex with the lowest binding energy was selected for molecular interaction analysis in Discovery Studio (BIOVIA, San Diego, CA, USA). The binding affinity (*K*_d_) and binding energy (Δ*G*) of compounds **4d** and **4f** towards Bcl-2 were evaluated using the following relation [47]

$$∆G=-RTlnK_{d}$$

where, *R* and *T* are the universal gas constant (1.987 kcal mol^−1^ K^−1^) and temperature (298 K), respectively.

#### 3.3.7. Statistical Analysis

OriginPro 8.5 software was used to conduct the statistical analysis. The mean and standard deviation of the data were displayed. Student’s *t*-test was used to examine differences, and *p* < 0.05 was regarded statistically significant. * *p* < 0.05, ** *p* < 0.01, *** *p* < 0.001.

## 4. Conclusions

Synthesizing a novel quinazoline incorporating substituted sulfonamide moieties in order to obtain anticancer activity with lower toxicity has attracted attention on the beneficial effect of this group to overcome one of the most serious problems in cancer chemotherapy, which is the lower efficacy and higher toxicity. Therefore, we aimed in this study to synthesize a novel series of quinazolinone derivatives carrying biologically active substituted-sulfonamide moieties at 3-position to achieve new compounds with the anticancer effect. Herein, a novel unexpected series of quinazoline incorporating substituted sulfonamide moieties was synthesized and evaluated in vitro for their anticancer activity. Some compounds were found to exhibit high cytotoxicity in the MCF-7 breast cancer cell line. Compounds **4d** and **4f** were appeared as the most effective derivatives against MCF-7 cells. Flow cytometry data showed that compounds **4d** and **4f** successfully triggered apoptosis in MCF-7 cells. Furthermore, changes in the expression of apoptosis-related markers at the gene and protein level were also indicative of apoptotic activity. Molecular docking of compounds **4d** and **4f** also suggested that they possess good binding affinity for Bcl-2. Overall, screening of the tested compounds could offer an encouraging framework in this field, which may lead to develop more potent, selective, clinically useful analogues anticancer agents. Furthermore, the existing compounds are currently under investigations for their in vivo anti-cancer activity in different transplantable mouse tumors model. A series of toxicity studies are also underway to determine the safety of these existing compounds. 

## Figures and Tables

**Table 1:** Cytotoxic activity, expressed as IC_50_, of synthetic compounds **4a**–**n** and doxorubicin against different cancer cell lines.

Compound No.	Cell Lines and IC_50_ (µM)				
	A549	HepG2	LoVo	MCF-7	HUVEC
**4a**	19.6 ± 1.7	35 ± 1.2	37.5 ± 1.8	14.2 ± 0.4	20.9 ± 1
**4b**	11.36 ± 1.6	33.7 ± 0.8	35.75 ± 1	36.2 ± 2	32.7 ± 1.2
**4c**	-	-	-	-	-
**4d**	6.6 ± 0.22	9 ± 0.3	6.87 ± 0.62	2.5 ± 0.2	7.8 ± 0.6
**4e**	-	-	-	-	-
**4f**	10.14 ± 0.24	11.7 ± 2	9.76 ± 1.68	5 ± 0.24	17.9 ± 0.6
**4g**	-	-	-	-	-
**4h**	13.3 ± 0.85	25.4 ± 0.95	31.7 ± 1	31.62 ± 1.3	24.3 ± 0. 56
**4i**	13.8 ± 0.5	20.6 ± 1.6	22.04 ± 1.01	26.4 ± 0.79	18.2 ± 2.2
**4j**	-	-	-	-	-
**4k**	-	-	-	-	-
**4l**	-	-	-	-	-
**4m**	-	-	-	-	-
**4n**	-	-	-	-	-
Doxorubicin	1.1 ± 0.01	2.8 ± 0. 01	1.5 ± 0.05	1.4 ± 0.3	3.7 ± 0.2

(-) Inactive at 40 µM (highest concentration tested). The values represented as the average ± SD (*n* = 3).

**Table 2:** Molecular docking of compound **4d** and **4f** with Bcl-2.

Donor Atoms	Acceptor Atom	Distance (Å)	Type of Interaction	Binding Free Energy, Δ*G* (kcal mol^−1^)	Binding Affinity, *K*_d_ (M^−1^)
Bcl-2 and DRO *					
LIG:N ASP70:OD2 MET74:CE LEU96:CD1 LIG:C PHE63 PHE71 LIG:C LIG LIG LIG LIG LIG	ASP70:OD2 LIG LIG LIG TYR67 LIG LIG LEU96 ARG105 ALA108 LEU96 ALA108 VAL92	2.9553 4.8135 3.5674 3.8681 3.5199 5.2880 4.9682 4.9539 5.2941 4.4414 5.1674 4.4935 5.0873	Conventional Hydrogen Bond Electrostatic (Pi-Anion) Hydrophobic (Pi-Sigma) Hydrophobic (Pi-Sigma) Hydrophobic (Pi-Sigma) Hydrophobic (Pi-Pi T-shaped) Hydrophobic (Pi-Pi T-shaped) Hydrophobic (Alkyl) Hydrophobic (Pi-Alkyl) Hydrophobic (Pi-Alkyl) Hydrophobic (Pi-Alkyl) Hydrophobic (Pi-Alkyl) Hydrophobic (Pi-Alkyl)	−10.2	3.03 × 10^7^
Bcl-2 and Compound (**4d**)					
LIG: H MET74:CE LEU96:CD1 LIG:C TYR67 ALA108 LIG:C LIG	GLU95:OE1 LIG LIG PHE71 LIG LIG:C MET74 VAL92	2.0244 3.4311 3.9269 3.6605 3.8844 3.7959 4.8241 5.3864	Conventional Hydrogen Bond Hydrophobic (Pi-Sigma) Hydrophobic (Pi-Sigma) Hydrophobic (Pi-Sigma) Hydrophobic (Pi-Pi Stacked) Hydrophobic (Alkyl) Hydrophobic (Alkyl) Hydrophobic (Pi-Alkyl)	−7.8	5.25 × 10^5^
Bcl-2 and Compound (**4f**)					
TYR67:HH PHE63 TYR67 GLU95:C,O;LEU96:N ALA108 LIG:C LIG:C LIG LIG	LIG:O LIG LIG LIG LIG:C LEU96 MET74 ARG105 ALA108	2.4126 4.8823 5.1361 3.9675 3.7719 4.6857 4.0247 5.4994 5.1493	Conventional Hydrogen Bond Hydrophobic (Pi-Pi T-shaped) Hydrophobic (Pi-Pi T-shaped) Hydrophobic (Amide-Pi Stacked) Hydrophobic (Alkyl) Hydrophobic (Alkyl) Hydrophobic (Alkyl) Hydrophobic (Pi-Alkyl) Hydrophobic (Pi-Alkyl)	−7.5	3.17 × 10^5^

* Chemically DRO is 1-(2-{[(3S)-3-(aminomethyl)-3,4-dihydroisoquinolin-2(1H)-yl]carbonyl}phenyl)-4-chloro-5-methyl-N,N-diphenyl-1H-pyrazole-3-carboxamide.

## Data Availability

The data presented in this study are available on request from the corresponding author.

## Supplementary Materials

The following are available online, Figure S1: Validation of docking protocol by redocking the ligand present in the crystal structure and comparing RMSD between the docking pose and crystal structure pose.
