# Supplementary material for: The Antiproliferative and Apoptotic Effects of a Novel Quinazoline Carrying Substituted-Sulfonamides: In Vitro and Molecular Docking Study
Source: Molecules. 2022 Feb 1;27(3):981. doi: 10.3390/molecules27030981 (PMC8838787; doi:10.3390/molecules27030981)
Supplement: Supplementary file 1 [file molecules-27-00981-s001.zip › molecules-1535381-supplementary.pdf]

Supplementary material

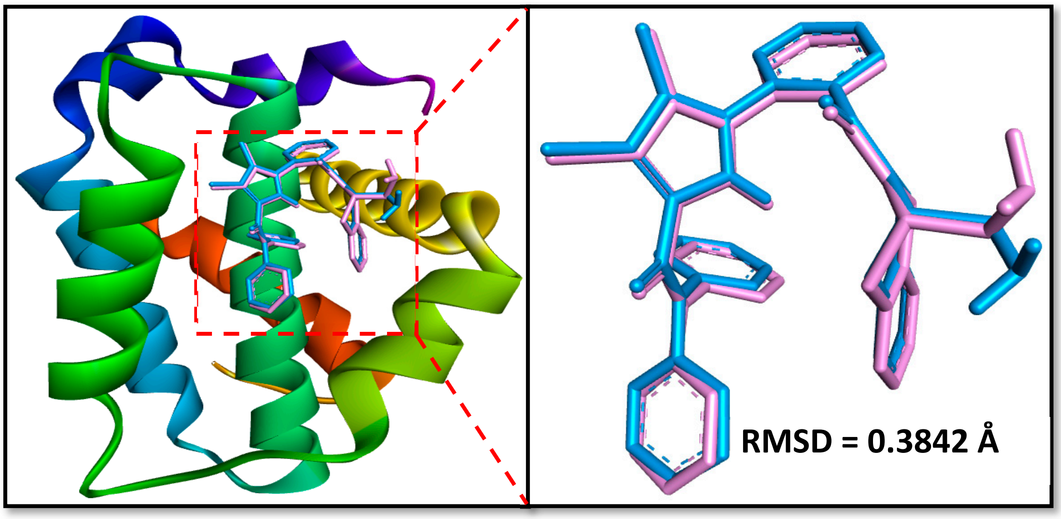

Figure S1: Validation of docking protocol by redocking the ligand present in the crystal structure and comparing RMSD between the docking pose and crystal structure pose.
